# Familial digit ratio (2D:4D) associations in a general population sample from Wales

**DOI:** 10.1016/j.earlhumdev.2017.06.006

**Published:** 2017-09

**Authors:** Gareth Richards, Wynford Bellin, William Davies

**Affiliations:** a Autism Research Centre, Department of Psychiatry, University of Cambridge, UK; b School of Social Sciences, Cardiff University, UK; c MRC Centre for Neuropsychiatric Genetics and Genomics, Division of Psychological Medicine and Clinical Neurosciences and Neuroscience and Mental Health Research Institute, Schools of Psychology and Medicine, Cardiff University, UK

**Keywords:** 2D:4D, Assortative mating, Digit ratio, Prenatal testosterone, Sex hormones, Sex linkage, Transgenerational effects

## Abstract

**Background:**

The relative length of the second and fourth fingers (2D:4D) may be a sex-linked correlate of prenatal androgen exposure. However, the nature of the sex-linkage is controversial, with evidence for both X- and Y-linkage of the 2D:4D phenotype.

**Aims:**

To examine transgenerational effects relating to sex-linkage. In addition, assortative mating on 2D:4D was considered, as well as associations between 2D:4D and age and sex.

**Study design:**

A family study was conducted. Parents and offspring completed a demographic questionnaire, and digit ratios were calculated from photocopies of participants' hands.

**Subjects:**

We recruited and phenotyped 585 individuals attending a cultural festival in Wales. 2D:4D information was available for 47 mother-son dyads, 70 mother-daughter dyads, 31 father-son dyads and 30 father-daughter dyads.

**Outcome measures:**

Correlations between 2D:4D of parents and children, as well as between mothers and fathers were conducted. 2D:4D was also examined in relation to age and sex.

**Results and conclusions:**

There was a sex difference in 2D:4D (males < females). Within the dyads, there was a significant positive correlation between mother and daughter 2D:4D, but no significant correlation between mother and son ratios, nor between father and offspring ratios. The overall pattern of correlations (with emphasis on father-son dyads) was not supportive of Y-linkage. There was a positive correlation between 2D:4D and age in children, and a negative correlation between 2D:4D and age in adults, and no evidence of assortative mating. Our data are consistent with the notion of 2D:4D as a sexually-dimorphic, mildly age-sensitive, and transgenerationally-transmitted trait that is more likely to be X- than Y-linked.

## Highlights

• Digit ratio (2D:4D) may be a sex-linked correlate of prenatal sex hormone exposure.
• 2D:4D was examined in a family design study of a general population sample.
• Correlations for 2D:4D within parent-child dyads were more consistent with X- rather than Y-linkage.
• There were no significant correlations between 2D:4D of mothers and fathers.
• 2D:4D correlated positively with age in children, and negatively with age in adults.

## 1 Introduction

The ratio of lengths of the index (2D) and ring (4D) fingers (2D:4D) has traditionally been viewed as an indication of prenatal exposure to sex hormones, with lower ratios indexing relatively high exposure to androgens (notably testosterone) relative to oestrogens [1]; this view is consistent with the robust observation that 2D:4D ratio is, on average, lower in males than in females [2], [3]. Recent analyses have suggested that an individual's 2D:4D ratio may not only be influenced by the degree of exposure to these hormones, but also by their sensitivity to them; moreover, as the 2D:4D ratio exhibits a degree of lability in the first two years of life, hormonal influences upon it may persist into the early postnatal period [4].

The relationship between 2D:4D ratio and age throughout life is currently somewhat uncertain: whilst some cross-sectional studies have provided evidence for an increasing digit ratio with age in children [5], and across a considerable range of the lifespan [6], others have reported negative associations in children [7] and young adults [8]. Alternatively, cross-sectional studies of children [3], [9] and adults [3] have indicated that there is no significant association. The discrepancy between these studies may potentially be explained by different study populations, by low power giving rise to false positive or false negative results, or by the assessment of populations with differential, or excessively-narrow, age limits. Longitudinal studies examining 2D:4D ratio in children by various means have indicated that the measure tends to increase slightly in both younger males and females with age, with more pronounced effects with regard to the left-hand, and null (or even opposite effects) in older children [10], [11], [12], [13].

Twin studies have indicated that the 2D:4D ratio may be partially influenced by genetic factors. For instance, Hiraishi et al. [14] provided evidence to suggest that an additive genetic influence could explain 60–70% of the phenotypic variability within the 2D:4D ratio, with the genetic influence being more pronounced for the left-hand. Family studies have provided conflicting accounts suggesting that 2D:4D might be X-linked [15] or Y-linked [16]. The first of these studies, Phelps [16], measured finger lengths directly from 20 US families, using a hand-measuring board, as well as from outline drawings of the left hand. Rather than calculating 2D:4D, this study divided participants into three phenotypes (2D > 4D, 2D = 4D, and 2D < 4D) (no differences in categorisation between the two methods were found). Phelps suggested that the distribution of these phenotypes could be accounted for by the effects of an X-linked gene with two alleles together with some additional interaction effects from genes on the X chromosome. The second report, Voracek and Dressler [16], measured finger lengths from photocopies, and observed heritability estimates of 57% and 48% for the right and left hands, respectively. This study reported significant correlations between paternal 2D:4D and that of sons (*r* = 0.305–0.363) and daughters (*r* = 0.131–0.276), as well as significant correlations between maternal 2D:4D and that of sons (*r* = 0.194–0.328) and daughters (*r* = 0.293–0.299). Correlations across male family members (*r* = 0.305–0.543) appeared to be stronger than those across female family members (*r* = 0.217–0.299), indicative of a Y-linked genetic effect.

Comparing the findings of Phelps [15] and Voracek and Dressler [16] is problematic because of their considerable differences in methodology. Unfortunately, two further reports [17], [18] relating to the heritability of 2D:4D have failed to provide a clearer picture. Whilst Ventura et al. [17] identified a significant positive correlation between the 2D:4D ratio in mothers and the same measure in their newborn daughters (*r* = 0.28–0.35) they did not find a significant association between maternal and newborn son 2D:4D (*r* = − 0.03-0.227); statistical comparison of these two correlations suggests no significant difference. Likewise, although Ramesh and Murty [18] reported 40–70% additive genetic variance in relation to distal extent of 2D and 4D, the study did not find a sex difference in digit ratio, nor any evidence of sex linkage.

In addition to being partially heritable, the 2D:4D ratio may represent a surrogate marker of assortative mating: Voracek, Dressler, and Manning [19] reported that 2D:4D for both hands were weakly positively correlated (*r* = 0.18–0.19) within couples, a result consistent with the notion that individuals who experience similar prenatal environments may be attracted to one another. However, although Manning [20] reported a similar weak positive correlation (for mean 2D:4D of the right and left hand) in 221 couples from Liverpool, UK (*β* = .15, *p* = .03), Hauth et al. [21] observed no significant correlations between the 2D:4D ratios of parents of typically developing children or parents of children with autism spectrum conditions. Further to this, Ramesh and Murty [18] reported no evidence of assortative mating on 2D:4D in a sample of 148 father-mother dyads from India.

In the present exploratory study, we collected 2D:4D data on a moderately-sized sample of participants (parents and children) from the general population recruited at a Welsh cultural festival. The study had four main aims: i) to confirm the well-established sex difference for the 2D:4D ratio, ii) to examine possible relationships between digit ratio and age, iii) to test for relationships between 2D:4D in mothers and their male and female offspring, and fathers and their male and female offspring, and iv) to test for relationships between 2D:4D ratios in partners with one or more shared children.

## 2 Material and methods

### 2.1 Participants

Members of the general population were recruited and tested at a stand held by the British Psychological Society's (BPS) Welsh Branch at the National Eisteddfod in Abergavenny, South Wales, between July 30th and August 6th 2016. Participants volunteered to take part in the study and were not provided with any monetary or other incentive.

### 2.2 Procedure

The current research was reviewed by Cardiff University School of Psychology Ethics Committee (ethical clearance number: EC.16.06.14.4538R), and was conducted in accordance with the Declaration of Helsinki. After reading an Information Sheet, adult participants provided written consent to continue with the study, whilst written parental assent (as well as child consent) was obtained for minors to participate. A brief demographic questionnaire was then used to obtain information on participants' sex, age, ethnicity, and relationship to anyone else participating in the study. A Hewlett Packard ENVY 4500 portable photocopier was used to scan participants' left and right hands, and greyscale images (optimised for quality rather than scanning speed) were made at a resolution of 300 ppi. These images were saved, and 2D:4D ratios were subsequently calculated for each hand using AutoMetric 2.2 for Windows [22]. Finally, participants were provided with a Debrief Sheet, and were given the opportunity to ask questions about the study. All study documentation was available in both English and Welsh languages.

### 2.3 Analysis and statistics

2D:4D ratios were calculated twice (several weeks apart, by the same researcher), and intra-class correlation coefficients (two-way mixed, single measures with absolute agreement) were conducted to analyse reliability of measurement. Repeatability was determined to be high for both R2D:4D, *ICC* = 0.929, *F* = 28.47, *p* < 0.001, and L2D:4D, *ICC* = 0.917, *F* = 23.192, *p* < 0.001. Mean R2D:4D and L2D:4D values were computed from these two sets of measurements, and were used in all subsequent analyses.

As 2D:4D ratios were not normally-distributed (as indexed by Shapiro-Wilk test), between-group comparisons were analysed using one or two-tailed Mann-Whitney *U* test as appropriate, and correlations were analysed using Spearman's test. For ease of comparison with findings from other studies, and because they are relatively robust to deviations from normality, correlation coefficients obtained by Pearson's test are also reported. Although very few notable differences are observed between the parametric and non-parametric analyses, interpretations of the findings are based upon non-parametric tests unless otherwise stated. The strength of correlations is compared statistically using Fisher's r-to-z transformation. *p*-Values < 0.05 were considered as being nominally significant; as the study was largely exploratory in nature, no corrections were made to take into account multiple testing issues. Data are presented as mean values (*M*) with standard deviation (*SD*) of the mean.

## 3 Results

### 3.1 Demographics

585 participants (340 females) of predominantly White European ethnicity (97.4%) and aged 5-89 yrs (*M* = 40.14, *SD* = 22.77) completed the study. 2D:4D ratio information was obtained for 47 mother-son dyads, 70 mother-daughter dyads, 31 father-son dyads and 30 father-daughter dyads; information was available for 17 father-mother-son triads, and for 17 father-mother-daughter triads.

### 3.2 Relationship between R2D:4D and L2D:4D

There was a highly significant positive correlation between R2D:4D and L2D:4D across the total sample, *rho* (578) = 0.636, *p* < 0.001 (*r* = 0.644, *p* < 0.001), as well as within males, *rho* (234) = 0.626, *p* < 0.001 (*r* = 0.636, *p* < 0.001) and females separately, *rho* (331) = 0.617, *p* < 0.001 (*r* = 0.631, *p* < 0.001). According to generally accepted criteria, the magnitude of these correlations was strong (Cohen [23]; *rho* < 0.3 = weak, 0.3–0.49 = moderate, ≥ 0.5 = strong).

### 3.3 Relationships between 2D:4D ratio and sex

Across the overall sample, as expected, there were highly significant effects of sex on R2D:4D (males, *M* = 0.955, *SD* = 0.033; females, *M* = 0.968, *SD* = 0.031, *U* = 30,249.5, *p* < 0.001) and L2D:4D (males, *M* = 0.954, *SD* = 0.037; females, *M* = 0.966, *SD* = 0.034, *U* = 31,662.5, *p* < 0.001). Fathers (*n* = 50) had significantly lower R2D:4D ratios than mothers (*n* = 106) (*M* = 0.953, *SD* = 0.031 vs. *M* = 0.968, *SD* = 0.032 respectively, *Z* = − 2.979, *p* = 0.003), and the same pattern of results was obtained for L2D:4D (*M* = 0.953, *SD* = 0.043 vs. *M* = 0.974, *SD* = 0.035, *Z* = − 3.052, *p* = 0.002). Male offspring (*n* = 61) had significantly lower R2D:4D ratios than female offspring (*n* = 83) (*M* = 0.958, *SD* = 0.025 vs. *M* = 0.966, *SD* = 0.031, one-tailed *U* = 2110.5, *p* = 0.0445), whilst a similar trend for L2D:4D was not significant (*M* = 0.954, *SD* = 0.034 vs. *M* = 0.958, *SD* = 0.036, one-tailed *U* = 2354, *p* = 0.473). R2D:4D was significantly higher than L2D:4D in females (*M* = 0.969, *SD* = 0.031 vs. *M* = 0.966, *SD* = 0.034, *Z* = − 2.266, *p* = 0.023), although no such effect was observed in males (*M* = 0.955, *SD* = 0.033 vs. *M* = 0.954, *SD* = 0.037, *Z* = − 0.756, *p* = 0.449).

### 3.4 Relationships between age and 2D:4D ratio

As the 2D:4D ratio may increase in early childhood and through adolescence, a novel approach was taken to examining relationships between digit ratio and age: correlations were conducted by sex for the whole sample (all ages), but also separately for adults (≥ 18 yrs) and children (≤ 17 yrs). Although the effects were not statistically significant in the overall sample, we noted an interesting dissociation by which significant positive correlations between age and 2D:4D ratio were observed for the right hand of boys and the left hand of girls, and significant negative correlations between age and 2D:4D ratio were observed in adult males (but not females) (see Table 1). The observed significant correlations were weak to moderate in strength.

**Table 1:** Correlations between digit ratio and age.

		Total sample					Males					Females				
		*n*	Spearman's		Pearson's		*n*	Spearman's		Pearson's		*n*	Spearman's		Pearson's	
			*rho*	*p*	*r*	*p*		*rho*	*p*	*r*	*p*		*rho*	*p*	*r*	*p*
All ages	R2D:4D	568	− 0.074	0.08	− 0.091	0.03	235	− 0.111	0.09	− 0.111	0.089	333	− 0.047	0.39	− 0.063	0.253
	L2D:4D	568	0.021	0.615	0.02	0.638	236	− 0.027	0.683	− 0.016	0.808	332	0.063	0.254	0.063	0.25
≤ 17 years	R2D:4D	141	0.213	0.011	0.174	0.039	65	0.293	0.018	0.291	0.019	76	0.141	0.224	0.092	0.428
	L2D:4D	142	0.225	0.007	0.201	0.016	66	0.123	0.324	0.117	0.348	76	0.31	0.006	0.274	0.017
≥ 18 years	R2D:4D	427	− 0.17	< 0.001	− 0.178	< 0.001	170	− 0.225	0.003	− 0.197	0.01	257	− 0.102	0.103	− 0.12	0.055
	L2D:4D	426	− 0.125	0.01	− 0.118	0.015	170	− 0.159	0.038	− 0.131	0.09	256	− 0.071	0.257	− 0.066	0.296

Note. All correlations reported are two-tailed.

### 3.5 Relationships between parental and offspring 2D:4D measures

**Fig. 1:**
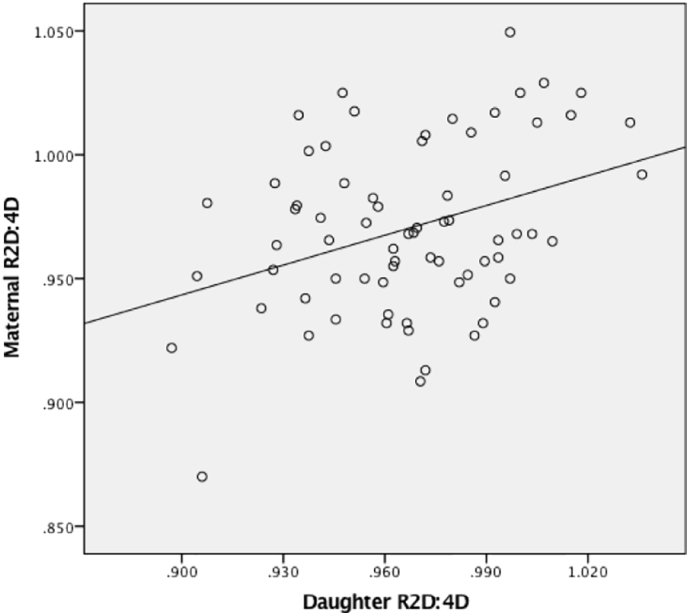
Spearman's correlation between maternal and daughter R2D:4D.

**Fig. 2:**
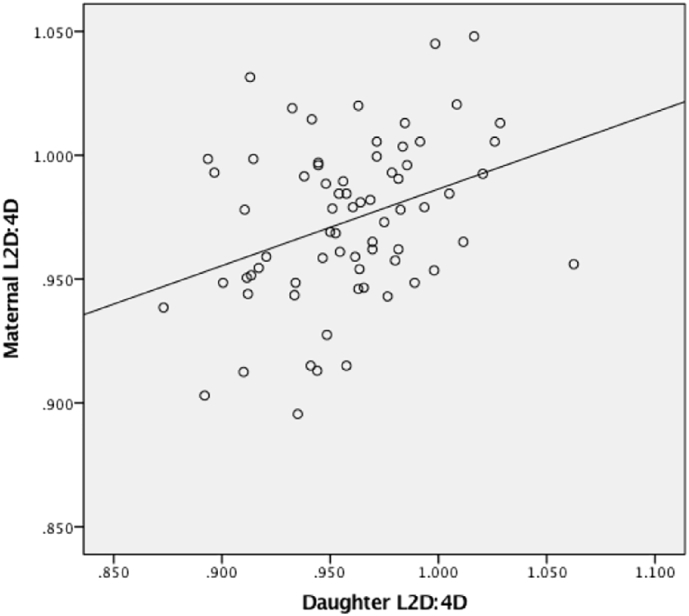
Spearman's correlation between maternal and daughter L2D:4D.

We found evidence for highly significant associations between maternal 2D:4D ratios and 2D:4D ratios across all offspring; when offspring were sub-divided by sex, it was clear that the initial observed association was primarily being driven by a positive correlation between maternal and daughter 2D:4D ratios (across both right and left hands, Fig. 1, Fig. 2 respectively) rather than a correlation between mother and son ratios (Table 2). Within our father-son and father-daughter dyads, we did not detect a significant association between paternal or offspring (of either sex) 2D:4D ratios. The observed statistically significant correlations were weak to moderate in strength.

**Table 2:** Correlations between parental and offspring 2D:4D.

		Total sample					Males					Females				
		*n*	Spearman's		Pearson's		*n*	Spearman's		Pearson's		*n*	Spearman's		Pearson's	
			*rho*	*p*	*r*	*p*		*rho*	*p*	*r*	*p*		*r*	*p*	*r*	*p*
Offspring R2D:4D	Mother R2D:4D	117	0.198	0.032	0.289	0.002	47	0.099	0.508	0.13	0.382	70	0.283	0.018	0.356	0.002
	Mother L2D:4D	117	0.256	0.005	0.26	0.005	47	0.218	0.141	0.221	0.136	70	0.28	0.019	0.28	0.019
	Father R2D:4D	60	0.037	0.776	0.01	0.941	31	− 0.231	0.211	− 0.193	0.299	29	0.276	0.148	0.179	0.354
	Father L2D:4D	61	− 0.128	0.327	− 0.116	0.372	31	− 0.052	0.782	− 0.169	0.362	30	− 0.151	0.425	− 0.084	0.659
Offspring L2D:4D	Mother R2D:4D	117	0.254	0.006	0.301	0.001	47	0.052	0.729	0.095	0.527	70	0.379	0.001	0.408	< 0.001
	Mother L2D:4D	117	0.261	0.004	0.28	0.002	47	0.151	0.31	0.182	0.221	70	0.351	0.003	0.347	0.003
	Father R2D:4D	60	0.044	0.738	0.059	0.652	31	0.089	0.635	0.099	0.597	29	− 0.005	0.979	0.013	0.948
	Father L2D:4D	61	− 0.064	0.623	− 0.005	0.967	31	0.058	0.756	0.119	0.523	30	− 0.171	0.365	− 0.071	0.709

Note. All correlations reported are two-tailed.

If X-linkage is present, we would expect the following differences in *rho* to be observed: (Father-Daughter = Mother-Son) > Mother-Daughter > Father-Son, with the last of these relationships most likely being negligible or nil [24]. However, if Y-linkage is present, we would expect the Father-Son correlation to be the strongest.

For R2D:4D, there was no significant difference between the slopes for Father-Daughter (*rho* = 0.276) and Mother-Son (*rho* = 0.099), *Z* = 0.74, *p* = 0.459. The slope for Mother-Daughter (*rho* = 0.283) was not significantly different from either the slope for Father-Daughter (*rho* = 0.276), *Z* = 0.03, *p* = 0.976, or Mother-Son (*rho* = 0.099), *Z* = 0.99, *p* = 0.322. However, the slopes were significantly different for Mother-Daughter (*rho* = 0.283) and Father-Son (*rho* = − 0.231), *Z* = 2.34, *p* = 0.019. As the former showed a positive correlation and the latter a negative correlation, no evidence was found for Y-linkage.

For L2D:4D, there was no difference between slopes for Father-Daughter (*rho* = − 0.171) and Mother-Son (*rho* = 0.151), *Z* = − 1.33, *p* = 0.184. However, although the Mother-Daughter (*rho* = 0.351) correlation did not differ from that of Mother-Son (*rho* = 0.151), *Z* = 1.11, *p* = 0.267, it was significantly different from Father-Daughter (*rho* = − 0.171), *Z* = 2.37, *p* = 0.018, the former being positive and the latter being negative. However, the slope for Mother-Daughter (*rho* = 0.351) did not differ significantly from that of Father-Son (*rho* = 0.058), *Z* = 1.37, *p* = 0.171.

Although Voracek and Dressler [16] compared the slopes observed in their data, they did not report whether the differences between them were statistically significant. For that reason, it was deemed useful to present this analysis here. For R2D:4D, the slopes for Father-Daughter (*r* = 0.276) and Mother-Son (*r* = 0.328) did not differ, *Z* = − 0.68, *p* = 0.497. Mother-Daughter (*r* = 0.293) did not differ significantly from Father-Daughter (*r* = 0.276), *Z* = 0.23, *p* = 0.818, or Mother-Son (*r* = 0.328), *Z* = − 0.49, *p* = 0.624. Mother-Daughter (*r* = 0.293) also did not differ from Father-Son (*r* = 0.363), *Z* = − 0.91, *p* = 0.363. For L2D:4D, Father-Daughter (*r* = 0.131) did not differ from Mother-Son (*r* = 0.194), *Z* = − 0.77, *p* = 0.441. Although Mother-Daughter (*r* = 0.299) was no different from Mother-Son (*r* = 0.194), *Z* = 1.41, *p* = 0.159, it was actually significantly stronger (positive) compared with Father-Daughter (*r* = 0.131), *Z* = 2.19, *p* = 0.029. Importantly, as with the analysis for R2D:4D, Mother-Daughter (*r* = 0.299) was not different from Father-Son (*r* = 0.305), *Z* = − 0.08, *p* = 0.936, so no strong evidence was provided for digit ratio being a Y-linked trait.

We tested to see whether the Father-Son correlations differed between the data presented in the current study and those of Voracek and Dressler [16] (for consistency with the analysis of Voracek and Dressler, *r* rather than *rho* values from our data are used). There was a significant difference for Father-Son correlations for R2D:4D between the current study (*r* = − 0.193) and Voracek and Dressler (*r* = 0.363), *Z* = − 2.87, *p* = 0.004, with the former observing a non-significant negative correlation, whist the latter observed a significant positive correlation. For L2D:4D the slope observed in the current study (*r* = 0.119) was not significantly different from that observed by Voracek and Dressler (*r* = 0.305), *Z* = − 0.97, *p* = 0.332.

### 3.6 2D:4D in mothers and fathers with at least one shared child

We did not identify any significant correlations between digit ratio of spouses for either hand: R2D:4D, *rho* (21) = − 0.207, *p* = .343 (*r* = − 0.306, *p* = .155); L2D:4D, *rho* (21) = − 0.171, *p* = .436 (*r* = − 0.156, *p* = 0.477). Furthermore, R2D:4D for mothers did not correlate with L2D:4D for fathers, *rho* (21) = − 0.083, *p* = 0.707 (*r* = − 0.081, *p* = 0.714), and R2D:4D for fathers did not correlate with L2D:4D for mothers, *rho* (21) = − 0.029, *p* = 0.897 (*r* = − 0.278, *p* = 0.199).

## 4 Discussion

Previous research has indicated that the 2D:4D ratio, an index of prenatal and/or early postnatal sex hormone exposure, differs significantly between sexes and by age, is correlated in parent-offspring dyads (notably mother-daughter), and may be a marker of assortative mating. Here, we attempted to examine these initial findings in a moderately-sized exploratory family-based study with participants recruited from the general population of Wales.

### 4.1 Sex differences in 2D:4D

We observed lower ratios in males than females for adults and younger participants, although the effect relating to L2D:4D in offspring was not statistically significant. The direction, and magnitude, of these findings is consistent with an extensive and robust previous literature [25], [26], as well as with the notion that males are exposed to higher levels of androgens relative to oestrogens than are females in utero and in early postnatal life. Although a previous meta-analysis [25] showed that the 2D:4D sex difference is larger with the right hand compared to the left, in the context of the current study, results were mixed with the L2D:4D ratio differing more between fathers and mothers, and the R2D:4D ratio differing more between sons and daughters.

### 4.2 Age-related effects

We found preliminary evidence for associations between 2D:4D and age, although these should be viewed cautiously in light of the absence of correction for multiple testing and the fact that digit ratio measurements were taken from scanned images and therefore may not completely recapitulate real-life measurements due to induced minor tissue distortion [27]. Specifically, we found a significant positive relationship between 2D:4D and age in individuals younger than 18 yrs, a finding consistent with data from previous longitudinal studies which have shown 2D:4D to increase during adolescence [10], [11], [12]. Additionally, we identified a significant negative correlation between 2D:4D and age in individuals aged 18 yrs or older, a result plausibly amenable to observation due to the fact that the current sample had a higher mean age than most published studies examining age effects on 2D:4D ratio. Our data are consistent with the idea that 2D:4D increases during childhood and adolescence, and then progressively decreases with ageing, and this interpretation might help to explain the inconsistencies in the literature relating to associations between 2D:4D and age. Potentially, given this result, age might routinely be co-varied for when examining relationships between digit ratio and psychological/physiological variables across a wide age range. Mechanisms which may feasibly underlie age-related changes in 2D:4D could include: fluctuations in circulating sex hormones, differential soft tissue growth and attrition in the ring and index fingers throughout life, modern influence of endocrine disruptors in the environment, and age-related changes in the ability to fully-extend the relevant digits for accurate measurement.

### 4.3 Transgenerational effects and assortative mating

Both left and right-hand maternal 2D:4D ratios correlated positively with left and right-hand 2D:4D ratios of their daughters (but not their sons), an observation which parallels the findings of Ventura et al. [17]. However, it is noted that other studies [15], [16], [18] showed both paternal and maternal 2D:4D ratios to correlate positively with those of sons and daughters. Although the current study does not provide strong evidence for X-linkage, the lack of significant correlations involving males potentially argues against Voracek and Dressler's [16] suggestion that 2D:4D is influenced by Y-linked loci. However, the present findings may also be explained by reduced power in those analyses (i.e. because fewer fathers and sons took part compared with mothers and daughters, respectively). Based upon the correlation coefficients of 0.305–0.363 in father-son dyads and 0.131–0.276 in father-daughter dyads reported by Voracek and Dressler [16], we would require a sample size of at least 57 father-son dyads and 101 father-daughter dyads to provide 80% power to detect a true effect with α = 0.05. Considering that the strength of correlations observed by Voracek and Dressler [16] appeared to differ between male and female family members, it is possible that 2D:4D is influenced by a complex interaction between shared genetic factors acting within mothers and their offspring and/or by other shared biological or environmental factors such as circulating maternal sex hormone levels to which the offspring would have been exposed in utero.

We found no evidence that 2D:4D ratios were correlated (either positively or negatively) between mothers and fathers with at least one shared child. This result is consistent with some previous research [18], [21], but is at odds with findings from two large European studies [19], [20], which indicated positive correlations between 2D:4D ratios of couples. These disparate findings suggest that any true effects, should they be present, are likely to be small; this area warrants further investigation with more highly-powered samples. In addition to this last point, future studies might benefit from using direct finger measurements, as photocopies/scans may be prone to minor distortions, which can result in slightly lower ratios [28].

Whilst our participant sample is of a reasonable size, and is somewhat representative of the general UK population, the present findings should be treated with an appropriate degree of caution. As many participants were unrelated to each other, and because considerably more mothers than fathers took part, some of our analyses are underpowered. Moreover, our sample may be biased by ascertainment being selected from festival attendees with an interest in science. There is some evidence to suggest that individuals (and family members) attracted to scientific pursuits may exhibit relatively high levels of autism-related traits [29], [30], [31], which may in turn be related to prenatal androgen exposure and the 2D:4D ratio [32], [33], [34].

In conclusion, our data provide further evidence that the 2D:4D ratio differs, on average, between sexes and throughout development, and that it is substantially influenced by trans-generational factors. The effects observed were more consistent with X- rather than Y-linkage. Although we did not demonstrate evidence for 2D:4D ratios being correlated between partners with a shared child (a possible index of assortative mating), inconsistent findings in the literature suggest that the topic warrants further investigation.

## Funding

This work was supported by a Student Research Grant from the European Human Behaviour and Evolution Association (EHBEA), which was awarded to GR. The work was partially undertaken within the Medical Research Council UK Centre for Neuropsychiatric Genetics and Genomics (MR/L010305/1). The funders played no role in study design, data collection, analysis and interpretation, writing of the manuscript, or the decision to submit the article for publication.

## Conflict of interest

None.

## Contributions

GR wrote the research proposal, designed the study, wrote the statistical analysis plan, collected and analysed the data, and drafted the initial manuscript. WD contributed to statistical analysis and interpretation, and WB translated English language questionnaires into Welsh. Both WD and WB revised the paper for important intellectual content. All authors read and approved the final article prior to submission.
